# 

**Published:** 1894-10

**Authors:** 


### Editorial.

### NOTICE.

Owing to a very serious illness of the Business Manager this issue of the journal was delayed. We hope our patrons will overlook the lateness of receiving their journal.

